# Photometric Monitoring of Electronic Cigarette Puff Topography

**DOI:** 10.3390/s23198220

**Published:** 2023-10-02

**Authors:** Keith Kolaczyk, Hao Jiang

**Affiliations:** Department of Biomedical Engineering, Lawrence Technological University, 21000 W 10 Mile Road, Southfield, MI 48075, USA; kkolaczyk@ltu.edu

**Keywords:** electronic cigarette, vaping, puff topography, aerosol, nicotine, e-liquid, atomizer, particulate matter, photometric sensor, pressure sensor

## Abstract

To study and monitor the adverse health consequences of using electronic cigarettes, a user’s puff topography, which are quantification parameters of the user’s vaping habits, plays a central role. In this work, we introduce a topography sensor to measure the mass of total particulate matter generated in every puff and to estimate the nicotine yield. The sensor is compact and low-cost, and is integrated into the electronic cigarette device to promptly and conveniently monitor the user’s daily puff topography. The topography sensor is comprised of a photometric sensor and a pressure sensor. The photometric sensor measures the mass concentration of the aerosol, based on scattering of near-infrared light from airborne particles, while the pressure sensor measures the flow rate. The topography sensor was tested under various conditions including a wide range of atomizer power, puff duration, and inhalation pressure. The sensor’s accuracy was validated by comparing the sensor’s readings with reference measurements, and the results matched closely with the trends reported by existing studies on electronic cigarettes. An example application for tracking a user’s puff topography was also demonstrated. Our topography sensor holds great promise in mitigating the health risks of vaping, and in promoting quality control of electronic cigarette products.

## 1. Introduction

Electronic cigarettes (“e-cigarettes” or “e-cigs”) have been rapidly growing in popularity in recent years, which has raised a great deal of concern about the health risks associated with vaping [1,2,3,4,5,6,7,8,9,10,11,12,13]. A considerable amount of studies have reported carcinogenic compounds, and trace metals in e-cigarette aerosols [7,8,9,12,14,15,16,17,18,19,20,21,22]. In some cases, the concentrations of these harmful contents are even higher in e-cigarette aerosols than in traditional cigarette smoke [7,14,15,18,19]. Inhalation of these chemicals has been associated with the development of multiple negative health conditions, including but not limited to heart disease, lung cancer, stenosis, asthma, and hypertension [7,8,9,20]. The health risks of vaping have been reported to be even more prevalent among youth and children [3,5,7,9,20,23,24].

In e-cigarette aerosol, the actual amounts of various chemicals and the particle size distributions of airborne particles, known as particulate matter (PM), are determined by two main aspects: the e-cigarette device, including hardware and e-liquid, and the user’s vaping habits. The aerosols generated depend on the type of e-cigarette device (such as disposable, rechargeable, cartridge, pod, mod, etc.), e-liquid compositions (including propylene glycol, vegetable glycerin, flavoring agents, and nicotine), and the e-cigarette brand/manufacturer [5,8,9,16,17,25,26,27,28]. E-cigarettes are often believed to be less lethal than combustible tobacco and are considered beneficial for cessation of traditional smoking. One study showed that the levels of certain carcinogens and toxicants in e-cigarette aerosols can be one to two orders of magnitude lower than those in traditional cigarette smoke [15]. However, due to the diversity and variety of e-cigarette products and the lack of manufacturing standards and quality controls, the actual e-cigarette aerosols can vary widely [5]. Poorly manufactured e-cigarettes and e-liquids can generate even more harmful constituents than traditional cigarettes [1]. The regulation of e-cigarette products is currently seen as a significant challenge by government agencies, such as the U.S. Food and Drug Administration (FDA) [29].

Given a specific e-cigarette device and e-liquid, the e-cigarette aerosol properties and nicotine yields are further decided by the user’s personalized vaping habits, known as puff topography [18,25,30,31,32,33,34,35,36]. In general, a user’s e-cigarette puff topography can be quantified by parameters, such as puff numbers, puff frequencies, inter-puff intervals, puff durations, puff flow rates, and puff volumes [25,30,31,36]. Several studies have investigated the puff topography and nicotine intake of different e-cigarette user groups [30,31]. Korzun et al. studied the effects of flow rates on toxicants in e-cigarette aerosols and e-liquid consumption [37]. Floyd et al. also reported the effects of flow rates on e-cigarette outputs [35,36].

From a broader perspective, puff topography should further include the user’s preferred device operational conditions [36]. Atomizer coil power, coil resistance, and coil temperature are known to strongly influence the e-cigarette aerosol outputs [25,32,36,38,39,40]. Zhao et al. studied the variations in e-cigarette aerosols under different coil temperatures [32]. Farsalinos et al. reported changes in users’ puff topography due to different power settings [34]. Floyd et al. revealed the effects of atomizer coil power on particle size distribution and the ratio between finer and bigger particles [38]. Pourchez et al. studied the effects of atomizer coil power and e-liquid compositions on aerosol output [41]. Mulder et al. demonstrated that e-cigarette aerosol particle size distributions and nicotine yields are strongly dependent on both battery voltage and coil resistance [39]. Lechasseur et al. investigated the effects of coil temperature, coil power, and e-liquid types on particle size distribution and lung deposition from e-cigarette aerosols [40].

To better understand the long-term adverse health consequences of vaping, it is essential to measure and track the personalized puff topography for all e-cigarette users. The puff topographies of many e-cigarette users represent potentially harmful vaping behaviors. For example, certain e-cigarette users have strong nicotine cravings and tend to overheat the e-liquids, which can cause the users to inhale very harmful carcinogenic constituents produced from the thermal decomposition of e-liquids at temperatures that are too high [6]. If puff topography can be monitored closely, puff by puff, users can be warned about such device misuse, potentially leading to modifications in their vaping behaviors towards healthier habits. To mitigate the health risks of vaping, there is a high demand for topography sensors built inside e-cigarettes that can monitor the user’s daily puff topography. Such topography sensors must be compact, low-cost, and compatible with mainstream e-cigarettes. In existing e-cigarette devices, the puff number, puff frequency, inter-puff interval, and puff duration can all be attained by monitoring the activation of the atomizer. Some e-cigarette brands can also automatically measure the coil resistance and record the power setting of the coil. Built-in airflow and pressure sensors, which are quite common in many e-cigarettes, activate the atomizer when the user inhales [2,5]. Such sensors can be used to monitor the puff flow rate and puff volume [36].

In existing studies on e-cigarettes, the properties of generated e-cigarette aerosols have been treated as a consequence resulting from the user’s puff topography [18,25,30,31,32,33,34,35,36]. Due to the aforementioned diversity of e-cigarette products and variations in vaping habits, direct measurements on the properties of e-cigarette output can potentially provide more valuable and reliable information than other parameters. Existing research methods typically implemented professional aerosol instruments or specially engineered topography devices to measure e-cigarette aerosols. Fast mobility particle sizer (FMPS), scanning mobility particle sizer (SMPS), and multi-stage micro-orifice uniform deposit impactor (MOUDI) were often implemented to measure the particle size distribution in e-cigarette aerosols [18,26,32,38,39,40,42]. Liquid chromatography/mass spectrometry (LC/MS), gas chromatography/mass spectrometry (GC/MS), and high-performance liquid chromatography (HPLC) were popularly used for chemical analysis of e-cigarette aerosols [23,25,30,39]. There are also established protocols for studying e-cigarette aerosols, developed by the Cooperation Centre for Scientific Research Relative to Tobacco (CORESTA) [43]. All these mentioned research methods require sophisticated, expensive, and bulky analytical instruments, and they are limited to studying e-cigarette aerosols in laboratories only. Several portable topography devices have been demonstrated, which allow in situ investigation of puff topography and aerosol properties [31,36,44]. Dunkhorst et al. implemented laser light polarization ratio method to measure the mass median diameter (MMD) of e-cigarette aerosols [44]. Floyd et al. developed a topography device based on Bernoulli flow cell to measure puff flow rate from differential pressure [36]. Although these topography devices opened up new possibilities to study puff topography in situ, they are not yet suitable to be integrated inside e-cigarettes for tracking users’ daily puff topography.

Due to the significance of e-cigarette aerosol properties, we propose further broadening the concept of puff topography to include quantification parameters on aerosol properties, if such parameters can be directly measured from the e-cigarette device itself. Considering its implications on the health risks of vaping, it is potentially more crucial than all other existing puff topography parameters. Previously, our group introduced the concept of “smart e-cigarettes” with built-in aerosol sensors [45]. Using a sensor assembly composed of a multi-wavelength photometric sensor and a gas sensor, the relevant aerosol properties, including the ratio between finer and bigger particles, the aerosol temperature, and the electrical resistance in response to volatile organic compounds (VOCs), were measured and tracked for every puff to analyze the user’s puff topography [45]. In this article, we introduce a new functionality of this topography sensor, which can measure the aerosol output—defined as the mass of total particulate matter (TPM) in each puff—and estimate the nicotine yield puff by puff. This “mass of TPM” is similar to the “mass of vaporized e-liquid (MVE)” or “mass loss per puff” found in the literature [35,46], and the differences will be discussed in later sections. In this article, we demonstrate the concept of our e-cig topography sensor, the construction of the prototype, the calibration and validation of the sensor’s responses, and the experimental results on various e-cigarette device settings and tracking one user’s puff topography.

The mass of TPM has been widely studied in association with puff topography. The majority of existing studies have implemented gravimetric approaches to measure e-liquid consumption by weighing the relevant e-cigarette components, such as the entire device, the cartomizer/clearomizer, and the filter pad for aerosol sample collections, before and after the puffs [6,18,25,30,34,35,37,38,39,46,47,48]. Several studies have directly measured the mass of TPM in the generated aerosols using analytical aerosol instruments [39,41,49]. Furthermore, external devices and experimental platforms have also been demonstrated. Wasisto et al. developed a piezoresistive cantilever sensor capable of measuring the mass of e-cigarette aerosols [50]. Dunkhorst et al. demonstrated the real-time monitoring of the PM mass concentration of e-cigarette aerosols using wavelength-dependent mid-infrared light extinction [51], and the polarization ratio method [44]. Wu et al. implemented photometric detections to measure e-cigarette aerosol concentrations using a laser beam in a scaled-model experiment [48]. These aforementioned examples are, by far, limited to in situ measurements of e-cigarette aerosols. It should be noted that none of the existing methodologies have enabled compact topography sensors to be built inside e-cigarettes.

## 2. E-Cig Topography Sensor Working Principle

**Figure 1:**
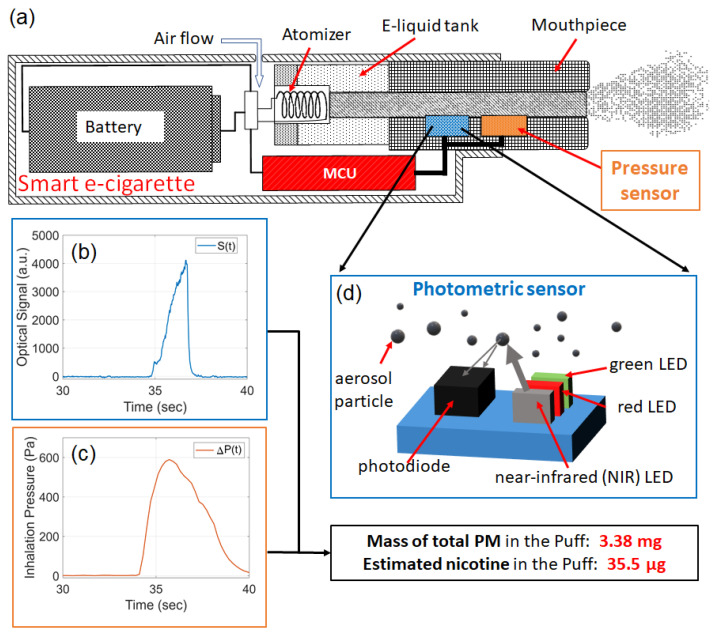
Working principle of the e-cig topography sensor. (**a**) Schematic of a “smart e-cigarette” equipped with a built-in topography sensor comprised of a photometric sensor, a pressure sensor, and a microcontroller unit (MCU). Example of (**b**) optical signal and (**c**) inhalation pressure collected from the photometric sensor and the pressure sensor, respectively. (**d**) Schematic of the photometric sensor for detecting an e-cigarette aerosol concentration.

Figure 1 shows the working principle of our e-cig topography sensor, which is integrated inside a smart e-cigarette device. A photometric sensor and a pressure sensor are both installed inside the aerosol delivery passage of the e-cigarette device to directly probe the aerosols from within the device. The purpose of the photometric sensor is to measure the real-time aerosol mass concentration, C(t). The function of the pressure sensor is to monitor the real-time volumetric aerosol flow rate, Q(t).

The mass of TPM in a puff, defined as $M_{puff}$, is given by:

(1) $$M_{puff}=\int C\left(t\right)Q\left(t\right)dt$$

where the integral is carried over the entire duration of the puff.

Photometric measurements for aerosols typically detect the intensity of light scattered from—or transmitting through—the aerosols, and the optical signal is then converted into a reading of the mass concentration of the PM according to a known calibration curve [44,48,51,52,53]. In this work, the photometric sensor, as schematically shown in Figure 1d, is comprised of multiple LEDs and a photodiode. We detect the aerosol concentration C(t) through the optical signal S(t) of the near-infrared light (wavelength centered at 880 nm) scattered from the high-concentration airborne particles in e-cigarette aerosols. The optical signal S(t) is proportional to the optical power transmitted through the active area of the photodiode. Since the size of the active area is a constant value, the optical signal is proportional to the intensity of the scattered light. Figure 1b shows an optical signal acquired from one puff. Consider the simplest scenario of the photometric measurement, C(t) is approximately proportional to S(t), i.e.,

(2) C(t)∝S(t)

The pressure sensor measures the real-time absolute air pressure, given as P(t), inside the aerosol delivery passage from a location between the atomizer and the mouthpiece. When a user draws a puff at the mouthpiece of the e-cigarette, a differential pressure, ΔP(t), referred to as “inhalation pressure” in this article, is applied between the air inlet and the mouthpiece to drive the flow of aerosol across the atomizer. Consider the pressure at the air inlet being equal to the ambient air pressure $P_{amb}$, the inhalation pressure $\Delta P\left(t\right)=P_{amb}-P\left(t\right)$. Figure 1c shows the inhalation pressure acquired from the same puff associated with the optical signal in Figure 1b. An e-cigarette aerosol is a pressure-driven turbulent flow and the flow rate is approximately proportional to the square root of the pressure drop [36,54]. For simplicity, if we ignore the effects of other factors, such as aerosol temperature, humidity, and concentration, the relationship between volumetric flow rate and inhalation pressure can be given as

(3) $$Q\left(t\right)\propto \sqrt{\Delta P(t)}$$

Based on the relations given in Equations (2) and (3), the mass of TPM in the puff ($M_{puff}$) in Equation (1) can be given by

(4) $$M_{puff}=\alpha \int S\left(t\right)\sqrt{\Delta P(t)}dt$$

where α is a coefficient to be determined by calibrating the sensor according to known references. Based on $M_{puff}$, the nicotine yield in the puff is estimated based on the weight concentration of nicotine in the e-liquid. For example, the sensor signals shown in Figure 1b,c have measured a mass of 3.38 mg of TPM and estimated a nicotine yield of 35.5 μg in the puff.

## 3. Materials and Methods

### 3.1. Prototype of E-Cig Topography Sensor and Smart E-Cigarette

**Figure 2:**
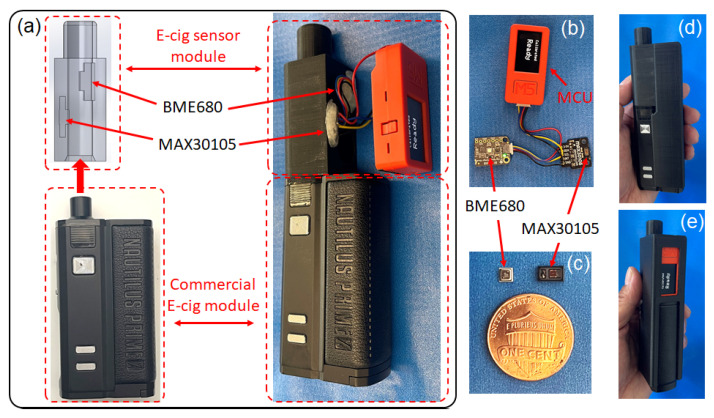
A prototype smart e-cigarette with an e-cig topography sensor. (**a**) Integration of the e-cig topography sensor assembly with a commercial e-cigarette module. (**b**) Photograph of the e-cig topography sensor assembly. (**c**) Photographs of the core components of the sensors, compared to the size of a U.S. penny coin. (**d**,**e**) Photographs of the constructed smart e-cigarette prototype.

Major components of our e-cig topography sensor assembly and a prototype of the smart e-cigarette are shown in Figure 2. For the photometric sensor, this work implements a commercial optical sensor MAX30105 (manufactured by Maxim Integrated), which was pre-soldered on a breakout board (manufactured by Pimoroni). For the pressure sensor, we use a commercial gas sensor, BME680 (manufactured by Bosch) pre-soldered on a breakout board (manufactured by Adafruit). BME680 sensors have been used to detect gas-phase components in indoor e-cigarette aerosols released into a room [55]. In previous work, we implemented the BME680 sensor to measure the temperatures and VOCs of e-cigarette aerosols [45]. The microcontroller unit (MCU) used in this work is a M5StickC PLUS (manufactured by M5STACK), comprised of an ESP32 microprocessor (manufactured by Espressif), a built-in battery, color display, push buttons, and a wireless communication module. The MCU was programmed using Arduino C. The MAX30105 and BME680 sensors were both connected with the MCU through an inter-integrated circuit (I2C) communication protocol. The MCU was connected to a lab computer via Bluetooth to communicate all the sensor data wirelessly. The sensor’s reading was displayed on the screen of the MCU and the detailed data analysis was carried out using a MATLAB script. A photograph of the sensor assembly is shown in Figure 2b, and the integrated smart e-cigarette device based on a commercial e-cigarette module is demonstrated in Figure 2a.

Figure 2a shows the commercial e-cigarette module used in this work, an Aspire Nautilus Prime X vaping mod, equipped with a BP clearomizer, comprising a BP sub-ohmic mesh coil. The labeled electrical resistance of the coil is 0.3 Ω, and the measured value is 0.29 Ω. The e-liquid used in this work is BB VAPES BRVND ENVY, with a labeled nicotine concentration of 11.75 mg/mL. The e-liquid has a labeled base material comprised of 70% vegetable glycerin (VG) and 30% propylene glycol (PG). The density of the e-liquid is about 1.12 g/mL, and the calculated weight concentration of nicotine is 1.05 wt%, accordingly, which was used in estimating nicotine yield from the mass of TPM. The housing and retaining frame of the e-cig topography sensor were designed with SOLIDWORKS and 3D printed using PLA plastic, as shown in Figure 2a. There are ports to house the sensor breakout boards to access the e-cigarette aerosol, and the aerosol passage at the center is connected to the mouthpiece of the e-cigarette module. All connections and ports were sealed with soft silicone to be air-tight. The constructed smart e-cigarette is larger than regular e-cigarette devices but is still sufficiently compact for a hand-held device, as shown in Figure 2d,e.

### 3.2. Sensor Signal Processing

Figure 1d shows the schematic of using the MAX30105 sensor for conducting the photometric measurement of an e-cigarette aerosol. The three LEDs emit light at center wavelengths of 527 nm (green LED), 660 nm (red LED), and 880 nm (near-infrared LED), respectively. They are switched on/off in an alternating fashion, such that only one color of light is detected by the photodiode at a time. The light absorbed in the active area of the photodiode generates a photocurrent, which is proportional to the optical power of light. The photocurrent is converted into a voltage signal, which is then digitized into an integer value using 14-bit analog-to-digital conversion. As a result, the output of the MAX30105 is a number that is proportional to the optical power absorbed by the photodiode. All three wavelengths were individually tested in our experiments and the results from the near-infrared wavelength performed the best. Therefore, in this work, only the near-infrared wavelength is used.

**Figure 3:**
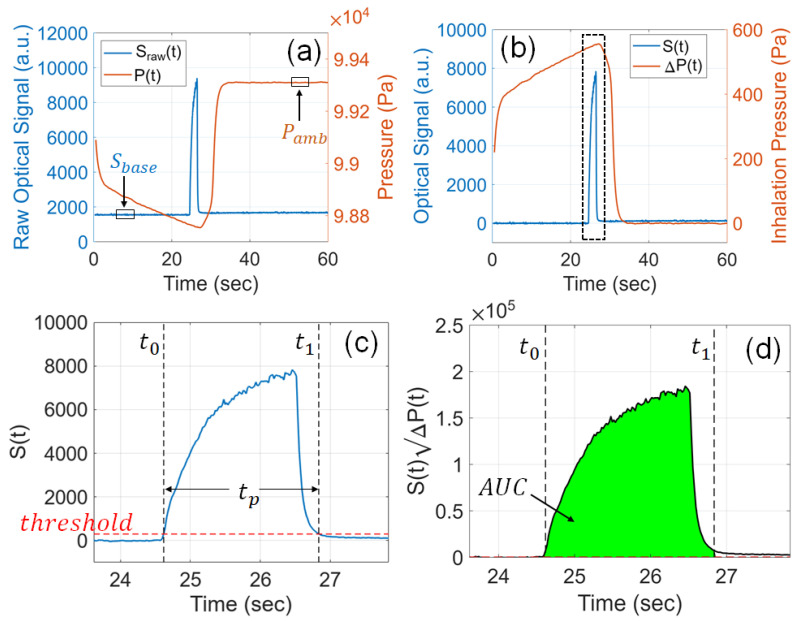
Algorithm for sensor signal processing. (**a**) Raw signals collected from the photometric sensor and the pressure sensor. (**b**) Baseline-subtracted optical signal and inhalation pressure. (**c**) Determination of the puff duration with a threshold on the optical signal. (**d**) Calculation of the numerical integral of $S\left(t\right)\sqrt{\Delta P(t)}$ during the identified puff.

Figure 3a shows one raw optical signal, $S_{raw}\left(t\right)$, acquired from the sensor when testing the smart e-cigarette with the vaping machine. The data sampling rate of the optical signal is 50 Hz, and the sampling interval is 20 ms, which is sufficient to resolve the rising and falling optical signal triggered by the generated e-cigarette aerosol. For all experiments conducted in this study, sensor signals were acquired over a duration of 60 s. This duration is much longer than the practical puff duration of a regular e-cigarette puff; however, it is necessary to synchronize the data acquisition sequence of the e-cig topography sensor with the control sequence of the vaping machine. At the beginning of the raw optical signal, there was no e-cigarette aerosol, and the baseline signal, $S_{base}$, was calculated by averaging $S_{raw}\left(t\right)$ during the window between t= 5 s and t= 10 s (marked in Figure 3a). The actual optical signal, S(t), shown in Figure 3b, was calculated by subtracting the baseline signal from the raw signal, i.e., $S\left(t\right)=S_{raw}\left(t\right)-S_{base}$, in order to correct the sensor’s response to zero aerosol.

Similarly, the signal from the pressure sensor, P(t), is also plotted in Figure 3a. The BME680 detects air pressure through piezoresistors, which can measure the mechanical stress or strain induced by a membrane displaced by the applied air pressure. The data sampling rate of the pressure signal is 5 Hz, and the sampling interval is 200 ms. Since the sampling rate for the pressure is lower than that for the optical signal, the acquired pressure signal is interpolated at the time steps of the optical signal. A negative pressure can be clearly observed when the aerosol is drawn out of the device. The ambient pressure, $P_{amb}$, is calculated by the average of P(t) during the window between t= 50 s and t= 55 s (marked in Figure 3a) after the pressure reaches the stable ambient pressure after the puff. The inhalation pressure, ΔP(t), calculated as $\Delta P\left(t\right)=P_{amb}-P\left(t\right)$, is plotted in Figure 3b.

The e-cigarette aerosol is detected only when S(t) is above a threshold, as illustrated in the magnified view of signals in Figure 3c. In this work, a threshold of 300 is applied in all signal processing. Each puff lasts from time stamp $t_{0}$ to $t_{1}$, which is located by the threshold. The puff duration is given by $t_{p}=t_{1}-t_{0}$. The puff duration measured in this approach is longer than the activation time of the atomizer because the aerosol still lasts for a brief duration after the atomizer begins to cool down. Our definition of puff duration is slightly different from that given by CORESTA [43], but the insights that can be derived from the measured values are the same.

In signal processing, the numerical calculation of the mass of TPM, according to Equation (4), is carried out as

(5) $$M_{puff}=\alpha \int_{t_{0}}^{t_{1}}S\left(t\right)\sqrt{\Delta P(t)}dt=\alpha AUC$$

where AUC is the area under the curve (numerical integral) of $S\left(t\right)\sqrt{\Delta P(t)}$, as plotted in Figure 3d.

For all experiments in this work, this algorithm of signal processing was implemented into the MCU of the e-cig topography sensor through the Arduino C code. The measurement results were automatically displayed on the screen of the smart e-cigarette after each acquisition. The same algorithm was also applied when post-processing the collected sensor data using the MATLAB script.

### 3.3. Vaping Machine and Reference Measurement

**Figure 4:**
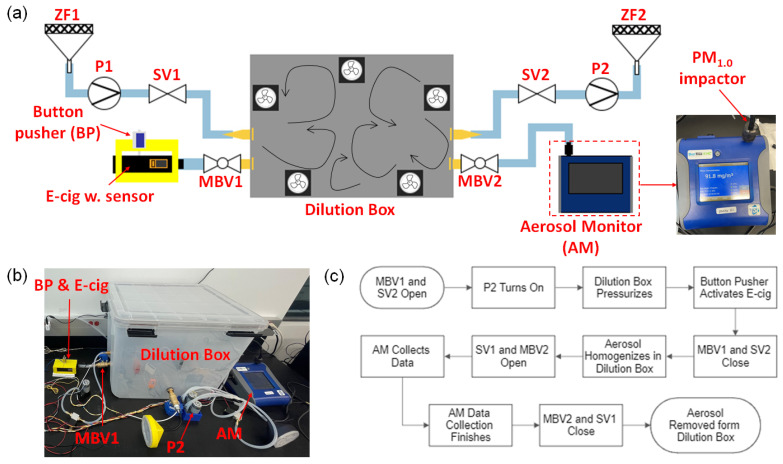
Experimental setup for reference measurements of the mass of TPM in the puff. (**a**) Schematic of the experimental setup. (**b**) Photograph of the constructed setup with a homemade vaping machine. (**c**) Flow chart of the control sequences used in each measurement of e-cigarette aerosols.

In order to calibrate and validate the response of our e-cig topography sensor, the mass of TPM in e-cigarette aerosols needs to be measured from both the e-cig topography sensor and a reference aerosol setup, simultaneously. In this work, we implemented a homemade vaping machine and a commercial aerosol monitor, DustTrak II 8530 (manufactured by TSI), in the reference measurement setup, as shown in Figure 4. The upper limit of the working range of the aerosol monitor (AM) is 400 mg/m${}^{3}$. Since the concentrations of e-cigarette aerosols are much higher than this limit, the generated e-cigarette aerosols must be diluted before the measurement. We implemented a dilution box, with a volume $V_{box}$ = 0.0946 m${}^{3}$, to dilute the aerosol by a factor ranging from 300 to 1000. For a given puff, all the PM in the generated e-cigarette aerosol was captured and homogenized inside the dilution box. The concept of our reference measurements based on aerosol dilution shares similarities with the experimental setup used by Sousan et al. [56]. Multiple fans were installed inside the dilution box to create chaotic flows to quickly homogenize the e-cigarette aerosol within 20 s, which was verified by readings from multiple miniature optical particle counters (PMSA003, manufactured by Plantower Technology) installed at different locations inside the dilution box.

The mass concentration of the diluted aerosol, $C_{AM}$, was measured by the aerosol monitor. Accordingly, the reference measurement result of the mass of TPM in each puff, $M_{Ref}$, is calculated by

(6) $$M_{Ref}=C_{AM}V_{box}$$

A PM${}_{1.0}$ impactor plate was installed at the inlet of the aerosol monitor to measure particles smaller than 1.0 μm. According to existing studies, the PM in e-cigarette aerosols, when evaluated by mass concentration, are mostly fine particles [26,39,40,41,42,56]. In our experiments, we tested both PM${}_{1.0}$ and PM${}_{2.5}$, and found that PM${}_{1.0}$ is at least 97% of PM${}_{2.5}$, which is consistent with the reported trends [42,56]. The aerosol monitor used in this work can detect particles as small as 0.1 μm. Therefore, our reference measurements can effectively measure the mass concentration of PM in the particle size range of 0.1 μm to 1.0 μm. Based on the reported results, our detection range can effectively cover the total mass of PM in e-cigarette aerosols [26,39,41,42,56]. Additional correction coefficients, which can be attained from gravimetric methods or more advanced aerosol instruments (such as SMPS), may be applied in our reference measurement results to further improve the accuracy. However, such improvements are expected to be marginal and the insights will not deviate from our findings based on this setup.

In our vaping machine, the switch button of the e-cigarette was automatically activated by a solenoid button pusher (BP) with 3D-printed housing to fix the smart e-cigarette and the button pusher together. All the valves, pumps, and the button pusher were automatically controlled through relays, which were further controlled by a programmed Arduino Mega2560 MCU. In order to minimize the loss of e-cigarette aerosols in the tubing and valves, motorized ball valves (MBVs), with a wide inner diameter (ID) of 0.5 inches, were used as valves to pass aerosols, such as MBV1 and MBV2 marked in Figure 4a. For valves passing clean air, regular solenoid valves (SVs) with narrow passages were used, such as SV1 and SV2. Air pumps, P1 and P2, were installed to drive the flow of clean air, which had been filtered with HEPA zero filters (ZFs), such as ZF1 and ZF2.

A photograph of the experimental setup is shown in Figure 4b, with key components marked in the photograph. Figure 4c shows a flow chart of the control sequence used for every reference measurement. Before the measurement, clean air was filled inside the dilution box through ZF1, P1, and SV1 to purge all PM out of the box. When an acquisition sequence was activated by the Arduino Mega2560 MCU, valves MBV1 and SV2 opened, and P2 turned on to pump the air out of the dilution box to create a negative pressure in the dilution box. This negative pressure serves as the inhalation pressure, mimicking a user’s inhalation. The peak inhalation pressure $\Delta P_{max}$ was controlled by the duration of pumping, $t_{vac}$, in this step. When $t_{vac}$ varied from 28 s to 112 s, $\Delta P_{max}$ changed from about 280 Pa to 600 Pa. Near the end of the pressurization, the button pusher was activated to push the switch button of the e-cigarette for the preset duration, $t_{btn}$. The generated e-cigarette aerosol was first measured by the e-cig topography sensor inside the e-cigarette device, and then passed through MBV1 to enter the dilution box. There was a delay of 1 s from the end of the button-pusher activation to the beginning of closing MBV1 to allow sufficient time to capture all generated aerosols in the dilution box. After all aerosol was captured inside the dilution box, MBV1 and SV2 closed and the aerosol was mixed with clean air to become homogenized in the box for 20 s. After the aerosol was homogenized in the dilution box, SV1 and MBV2 opened, and the aerosol monitor (AM) switched on to measure the mass concentration of the diluted aerosol at a flow rate of 3 L/min. After AM collected the data, valves MBV2 and SV1 closed, and the box was thoroughly cleaned by removing all aerosols inside the box. The cleanness of the air in the box was confirmed by the reading of particle counters inside the box. Then, the vaping machine was ready for the next measurement. After a few experiments, residues were observed on the inner wall of the box, however, these residues adsorbed on the wall will not affect the PM of the aerosol inside the box, which was also confirmed by the particle counters inside the box.

This control sequence for reference measurements was activated by the Arduino Mega2560 MCU while the data acquisition sequence of the e-cig topography sensor was triggered by the MCU on the sensor. In our experiments, these two sequences were started separately and manually. As a result, there can be a random time shift, up to 15 s, between the two sequences. Ideally, the aerosol should begin to appear in the optical signal around the same time stamp for all experiments. However, due to this artifact of a random time shift between two sequences, the signals can occur at a shifted time stamp, as can be seen from certain graphs of this article. To deal with this time shift, we acquired signals for a very long duration of 60 s for each puff, which can ensure that the sensor signals are completely collected when the aerosol is generated through the vaping machine during this long time frame. It should be noted that such a long duration of acquisition is only necessary for synchronizing the two sequences to compare the sensor’s reading with the reference measurement. For practical applications of the e-cig topography sensor alone inside an e-cigarette, the duration of the signal acquisition can be shortened to a few seconds or automatically adapted according to the user’s actual puff duration.

### 3.4. Sensor Calibration

The e-cig topography sensor’s response was calibrated according to the reference measurements. The goal of the calibration is to find a calibration coefficient, α, to make the sensor’s reading, $M_{puff}$, match the reference measurement, $M_{Ref}$. In our calibration experiments, the default configurations used for the smart e-cigarette and the vaping machine were as follows: 30 W atomizer power, 2.0 s button-pusher duration, and 54 s pressurization time for the dilution box. From each experiment, the calibration coefficient was calculated based on the following equations:

(7) $$M_{puff}=M_{Ref}$$

(8) $$\alpha =C_{AM}V_{box}/\int_{t_{0}}^{t_{1}}S\left(t\right)\sqrt{\Delta P(t)}dt$$

The experiments were repeated three times and the calibration coefficient was decided from the average of three trials. This coefficient was thereafter applied to the MCU of the e-cig topography sensor to directly measure the mass of TPM in each puff. The detailed results of calibration experiments are summarized in Table A2. For all experiments shown in this article, our sensor was calibrated twice. Before the testing on the atomizer power, puff duration, and inhalation pressure, the sensor was calibrated, and the attained coefficient, $\alpha_{1}$ = 3.5129 × 10${}^{-5}$, was applied to these tests. After running over 100 puffs with this setting, the BME680 sensor was removed from the device to clean the deposits of e-liquid on the sensor surface, and then re-installed into the device. It was found that the re-installed sensor had a slightly different airflow resistance and had to be calibrated again. The coefficient attained from the second calibration, $\alpha_{2}$ = 3.3322 × 10${}^{-5}$, was applied to the experiments for testing the cold atomizer coil and for tracking one user’s puff topography.

### 3.5. Sensor Validation

To validate the reading of our e-cig topography sensor and to study the effects of different device operating conditions and user vaping habits on the aerosol output, the smart e-cigarette was configured with various settings and tested in parallel with our reference measurement setup. The reading of the sensor, $M_{puff}$, was directly compared with the reference measurement, $M_{Ref}$, to evaluate the accuracy of our sensor. The effects of atomizer power, puff duration, and inhalation pressure, were individually experimented. The default settings for the smart e-cigarette and the vaping machine were set as follows: 30 W atomizer power, 2.0 s button-pusher duration, and 54 s pressurization time for the dilution box. For each set of experiments, one parameter of interest was changed while other parameters were kept at the default setting. Every given setting was tested three times. Statistical quantities (mean and standard deviations) were calculated from the results of the three trials and are presented in the plots with error bars. To study the e-cigarette aerosols in a steady state, the e-cigarette device was operated with six puffs to warm up the atomizer coil before each set of experiments, with an inter-puff interval of about 3.5 min. The experimental results from these tests are summarized in Table A3, Table A4 and Table A5.

### 3.6. Effects of Atomizer Power

**Figure 5:**
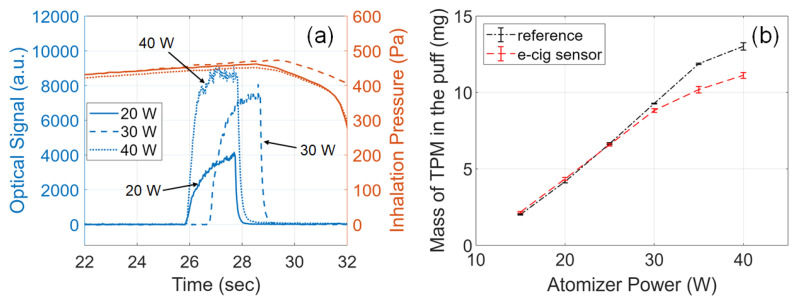
Effects of atomizer power on the e-cigarette aerosol output. (**a**) Example signals acquired from the e-cig topography sensor. Plots of sensor signals for different atomizer powers specified by line styles. Solid line: 20 W; dashed line: 30 W; dotted line: 40 W. (**b**) Mass of TPM in the puff measured from the e-cig sensor (red) and from the reference setup (black) versus atomizer power.

Atomizer power and coil resistance are two of the main factors influencing the PM generated by e-cigarettes. Studies have shown that higher powers produce aerosols with a greater mass of TPM per puff [34,38,39,40,41]. To study the effects of atomizer power on aerosol output, the e-cig topography sensor was tested by varying the atomizer power from 15 W to 40 W. Other conditions were kept at their default settings: 2.0 s button-pusher duration, 54 s pressurization time of the dilution box. Three experiments were carried out for each configuration; all results are plotted in Figure 5 and summarized in Table A3.

As shown in the curves plotted in Figure 5a, by using a higher atomizer power, a stronger optical signal was attained, which means an e-cigarette aerosol of a higher concentration. In addition, the transient details of the optical signal also suggest that with a higher atomizer power, the aerosol concentration grows to the peak at a faster rate, which is consistent with expectations, as a higher atomizer power gives a higher rate of thermal energy transfer to vaporize the e-liquid. For each condition, the relationship between the optical signal and inhalation pressure validates that our homemade vaping machine has consistent and precise controls on the button pusher and the inhalation pressure.

Figure 5b shows the mass of TPM measured from our e-cig topography sensor, compared with the reference measurements. Overall, the results of our sensor match very well with the trend of the reference measurements. For atomizer power lower than or equal to 30 W, the mass of TPM grows almost linearly with increasing atomizer power, which is consistent with the trend reported by Floyd et al. [38]. Particularly, in this range, the relative errors of individual measurements are mostly less than 9%. As listed in Table A3, only one data point, trial no. 3, has a relative error higher than 9%. For atomizer power above 30W, both the reference and e-cig topography sensor display a nonlinear relationship between the mass of TPM and the power, aligning with the logarithmic relationship reported by Pourchez et al. [41]. Within this range, the relative errors of the e-cig topography sensor have increased to 11–17%. We attribute this higher error to the optical signals from an aerosol concentration outside the calibrated range. The calibration of our sensor was essentially a single-point linear calibration, carried out at 30 W atomizer power, and the peak optical signal acquired during calibration was around 7500 (Table A2). When testing the atomizer power above 30 W, the peak optical signal reached higher than 8000 (Table A3), and the optical signal deviated from the linear dependence versus the aerosol concentration due to multiple scattering of light from high-concentration particles [53].

### 3.7. Effects of Puff Duration

**Figure 6:**
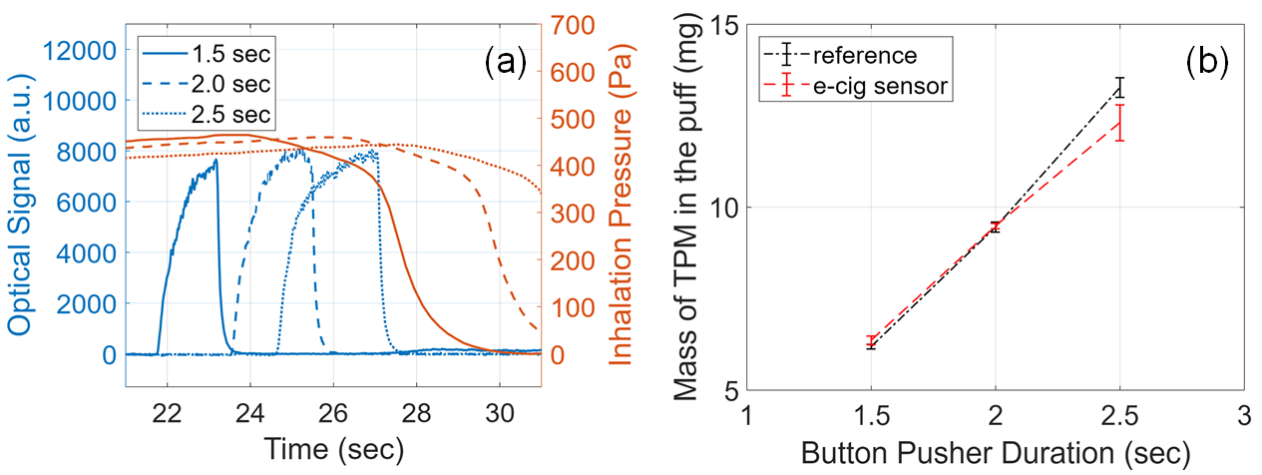
Effects of puff duration on the e-cigarette aerosol output. (**a**) Example signals acquired from the e-cig topography sensor. Plots of sensor signals for different button-pusher durations specified by line styles. Solid line: 1.5 s; dashed line: 2.0 s; dotted line: 2.5 s. (**b**) Mass of TPM in the puff measured from the e-cig topography sensor (red) and from the reference setup (black) versus button-pusher duration.

Puff duration affects the e-cigarette aerosol output significantly, as a longer puff duration can activate the atomizer for a longer period and generate more aerosol [26,32]. To study the effects of puff duration on the e-cigarette aerosol output, the e-cig topography sensor was tested by varying button-pusher durations from 1.5 s to 2.5 s. Other conditions were kept at the default settings: 30 W atomizer power and 54 s pressurization time of the dilution box. Three experiments were carried out for each configuration and all results are plotted in Figure 6 and summarized in Table A4.

As shown in Figure 6a, with a longer button-pusher duration, a longer puff duration can be clearly observed from the wider peak in the optical signal. From the data points plotted in Figure 6b, the mass of TPM almost grows linearly with longer button-pusher durations; this is consistent with existing studies [26,32]. The data listed in Table A4 show that the relative errors of measurements are mostly less than 6%. Only one data point for the 2.5 s button-pusher duration gives a relative error of about 11%. This increased error is again related to the higher aerosol concentrations when the button is pressed for a longer duration, as can be seen from the peak optical signal.

### 3.8. Effects of Inhalation Pressure

**Figure 7:**
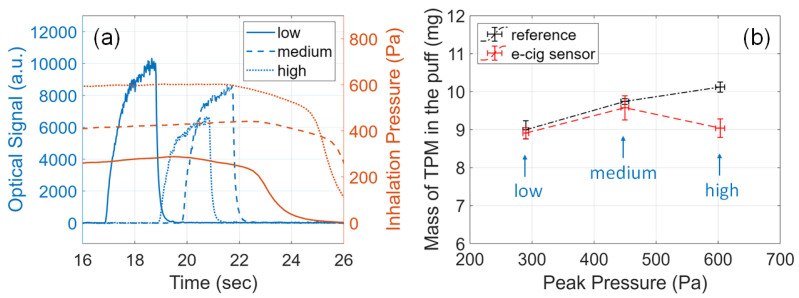
Effects of inhalation pressure on the e-cigarette aerosol output. (**a**) Example signals acquired from the e-cig topography sensor. Plots of sensor signals for different inhalation pressures specified by line styles. Solid line: low pressure (around 300 Pa); dashed line: medium pressure (around 450 Pa); dotted line: high pressure (around 600 Pa). (**b**) Mass of TPM in the puff measured from the e-cig topography sensor (red) and from the reference setup (black) versus peak inhalation pressure.

Given an e-cigarette device, the flow rate is determined by the inhalation pressure and the airflow resistance of the device [36,54]. To study the effects of flow rate on the e-cigarette aerosol output, the e-cig topography sensor was tested with varying inhalation pressures from 300 Pa to 600 Pa. This range was decided in order to match one user’s inhalation pressure when testing the smart e-cigarette device. The inhalation pressure was controlled through the aforementioned pressurization duration on the dilution box, from 28 s to 112 s. Under this range of testing conditions, the volumetric flow rate, measured using a flow meter installed inline with pump P2 in Figure 4a, was found to range roughly from 4.5 L/min to 7.5 L/min. Other conditions were kept at default settings: 30 W atomizer power and 2.0 s button-pusher duration. Three experiments were carried out for each configuration and all results are plotted in Figure 7 and summarized in Table A5.

As shown in Figure 7a, low pressure (around 300 Pa), medium pressure (around 450 Pa), and high pressure (around 600 Pa) were applied to draw the puffs from the e-cigarette mouthpiece. With a lower pressure and, therefore, a lower flow rate, the optical signal was stronger, which suggests a higher aerosol concentration, because the generated e-cigarette aerosol was extracted at a lower flow rate. From the reference measurements plotted in Figure 7b, the mass of TPM increases with higher pressure (higher flow rate), which matches the reported trends related to flow rate [35,37]. As shown in Figure 7b and Table A5, for low and medium pressure, the readings from our e-cig topography sensor align very closely with the reference measurements, maintaining a relative error below 7%. For high pressure (high flow rate), the relative error increased to 9–13% and the reading of our e-cig topography sensor deviated from the reference measurements. In this work, a simplified relationship between the flow rate and inhalation pressure, as seen in Equation (3), was used. For higher pressure, this equation requires further corrections to improve the accuracy, which can be achieved by more precise data fitting in the calibration procedure [36].

### 3.9. Effects of Cold Atomizer Coil

When an e-cigarette operates from a condition of a cold atomizer coil (at ambient temperature), referred to as the “initial state”, the aerosol output is significantly lower than that from a coil that has already been warmed up after a few puffs, known as the “steady-state” [6]. To study the effects of the atomizer condition, the smart e-cigarette was tested, starting from a condition when the atomizer coil was cold, and six puffs were tested continuously with inter-puff intervals of 3.5 min. The following setting was used for all six puffs: 30 W atomizer power, 1.5 s button-pusher duration, and 54 s pressurization time of the dilution box. To investigate the temperature-related trends, the aerosol temperature was also collected from the BME680 sensor, using the approach demonstrated in our previous work [45].

**Figure 8:**
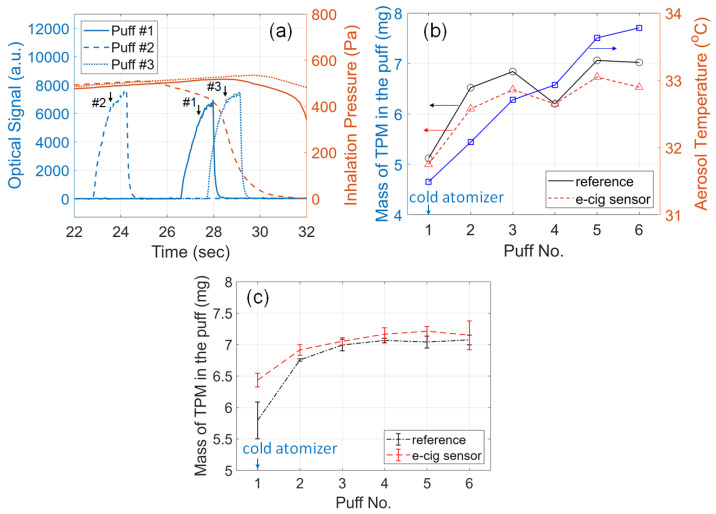
Effects of atomizer conditions (cold or warmed-up coil) on the e-cigarette aerosol output. (**a**) E-cig topography sensor signals acquired during the first three puffs from the smart e-cigarette starting to operate from a cold atomizer coil. The rising edges of the optical signals are marked with arrows. (**b**) Mass of TPM in the puff measured from the e-cig topography sensor (red triangles), from the reference setup (black circles), and aerosol temperature (blue squares), versus the puff number, as the coil was warmed up in one trial. (**c**) Mass of TPM versus the puff number as the coil is warmed up with experiments repeated in three cycles. Statistic quantities (mean and standard deviations), are plotted.

The sensor signals acquired from the first three puffs are shown in Figure 8a and the mass of TPM is given in Figure 8b. Starting from the cold atomizer coil, as more puffs were activated from the e-cigarette, the rising edges of the optical signals became steeper and reached higher amplitudes, as shown in the marked arrows. The mass of TPM in the first puff (for the cold atomizer) was noticeably lower than that of the second puff and so on. The aerosol temperature also increased as the atomizer was warmer with more puffs. Both the trend of aerosol output and temperature align with findings from reported studies [6]. The aerosol output reached a steady state after five or six puffs. Compared with the reference measurements, the e-cig topography sensor clearly captures the trends as the atomizer coil warms up. The relative error of the e-cig topography sensor’s reading is lower than 7%, at a level similar to the results in the aforementioned tests. To test the repeatability of the trend, the experiments were repeated in another three cycles and the results are plotted in Figure 8c. The trend of increasing e-cigarette aerosol PM output as the atomizer coil is warmed up can be clearly verified. For the cold atomizer (first puff), the sensor’s reading diverges more from the reference measurements compared to the subsequent puffs, probably due to the effects of air temperature inside the e-cigarette aerosol passage, affecting the optical sensor. The sensor was calibrated under steady-state conditions, where the optical sensor operated in a warmer environment. For the initial state (first puff), the temperature at the optical sensor was lower than that for the steady state (later puffs), which can cause the optical response to drift a little.

### 3.10. Tracking a User’s Puff Topography

**Figure 9:**
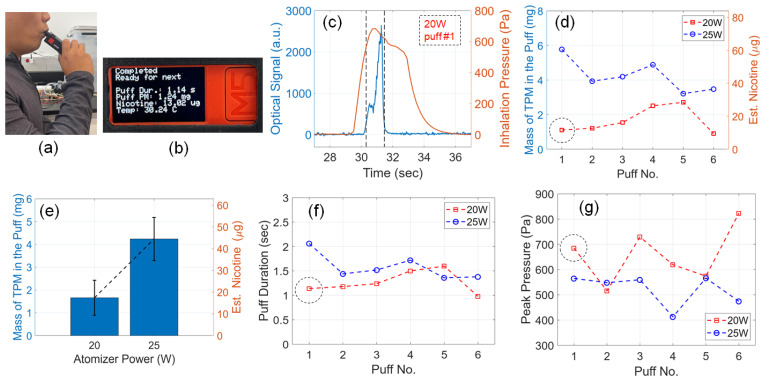
Tracking a user’s puff topography with the e-cig topography sensor. Photograph of (**a**) the user testing the smart e-cigarette and (**b**) the display on the device showing the results after one puff. (**c**) Sensor signals acquired during the first puff for 20 W atomizer power. The data points for this acquisition are marked as dashed circles on the curves in (**d**,**f**,**g**). (**d**) Mass of TPM in the puff and estimated nicotine yield from multiple puffs using 20 W and 25 W atomizer power. (**e**) Statistical quantity (mean and standard deviations) of the user’s puff topography for 20 W and 25 W atomizer power, respectively, attained from the puffs shown in (**d**). (**f**) Puff duration and (**g**) peak inhalation pressure acquired from the user’s puffs using 20 W and 25 W atomizer power.

One goal of the e-cig topography sensor is to measure the aerosol output and nicotine yield in each puff, regardless of the device setting of the e-cigarette, the atomizer condition, and the user’s vaping habits. As a proof of concept, the smart e-cigarette was directly tested by a user, one author of this article, with the user’s consent, using the user’s preferred settings of 20 W and 25 W atomizer power, as shown in Figure 9a. For each power setting, the user carried out six puffs with the user’s own comfortable puff duration and inhalation pressure. After each puff, the e-cig topography sensor’s reading was displayed on the device, showing the puff duration, the mass of TPM in the puff, the estimated nicotine yield, and the aerosol temperature, as shown in Figure 9b.

Figure 9c shows the sensor signals acquired for the first puff under 20 W atomizer power. It should be noted that the profiles of the signals are different from those acquired when driven by the vaping machine, because of the different fashions in applying the inhalation pressure from the user, compared with that from the vaping machine. Figure 9d shows the aerosol output and nicotine yield for all the puffs. The nicotine yield was estimated based on the weight concentration of nicotine in the e-liquid using the aforementioned approach. For 20 W and 25 W atomizer power, respectively, the statistical quantities of the attained six puffs are analyzed and plotted in Figure 9e. It can be clearly observed that when the user applied a higher atomizer power, the aerosol output increased significantly. Such a simplified statistical analysis can provide valuable information on the user’s puff topography. According to the puff duration and peak pressure plotted in Figure 9f,g, the aerosol output trends are consistent with the aforementioned results attained from the vaping machine, which validated the effectiveness of our e-cig topography sensor when tracking the user’s puff topography. One can observe that the parameters that are controlled by the same user, including the puff duration and peak pressure, can vary over a wide range, puff after puff. In these tests, the user simply took all puffs naturally and did not intend to change the vaping pattern. Despite these variations, using our e-cig topography sensor, the mass of TPM and nicotine yield were conveniently tracked for every puff, which provided crucial information for evaluating the health risks in the user’s vaping habits.

## 4. Discussion

Our e-cig topography sensor allows quantitative measurements of e-cigarette aerosols within the smart e-cigarette device, and such measurements have so far only been carried out in well-equipped laboratories or by using bulky topography devices. The results measured from our e-cig topography sensor matched well with the trends reported in existing studies, and the relative error is mostly lower than 9% for most of the trials. When examining all experimental results listed in Table A3, Table A4 and Table A5, the summation of the mass of TPM measured by the e-cig topography sensor from all puffs is 296.87 mg, while the reference measurement result is 314.48 mg, which suggests an overall relative error of about 5.60%. Considering the compactness and simplicity of the sensor, the achieved accuracy is remarkable. Based on the experimental results, our e-cig topography sensor has promptly, conveniently, and accurately monitored the mass of TPM, puff by puff, over a wide range of settings, including atomizer power, puff duration, and inhalation pressure; this has never been demonstrated from sensors integrated inside e-cigarettes before. The success of our topography sensor stems from the strategy of combining multi-parameter sensors for concurrent pressure and optical measurements, similar to the strategies demonstrated for other health-related sensor applications [57,58].

The capability of our e-cig topography sensor can open up new avenues to monitor all e-cigarette users’ daily puff topography and mitigate the health risks of vaping. With our e-cig topography sensor, the mass of TPM and nicotine yield in every puff can be closely tracked, regardless of the setting of the device, the condition of the atomizer, and the user’s inhalation fashion. In particular, on certain types of e-cigarette devices, atomizer power control does not exist, making it impossible to quantify how much particulate matter and nicotine can be inhaled by the user. Since our sensor directly measures the aerosols of the e-cigarette output, it can directly function with such e-cigarette devices to make quantitative measurements. From the perspective of regulation, our e-cig topography sensor can be used for the quality control of e-cigarette products by comparing the measured mass of TPM with the predetermined ideal values for a given setting. When significant differences are detected, the user should be warned about the potentially malfunctioned device. For example, when the e-liquid is nearly empty in the tank and the atomizer is relatively dry, the mass of TPM in the generated e-cigarette aerosols will be different from normal. With our sensor, such a condition can be detected and the user will be notified. In addition, our e-cig topography sensor can also allow the nicotine dose to be tracked for every puff. Such a feature can be very useful for special e-cigarette devices designed for the cessation of smoking traditional tobacco.

In our work, the photometric measurement of the aerosol concentration is based on light scattering, and the mass concentration of aerosol is proportional to the intensity of scattered light, as illustrated in Figure 1. Alternatively, the aerosol concentration can also be measured using a light transmission configuration, in which the concentration is proportional to the absorbance based on Beer–Lambert’s Law. The transmission scheme will be investigated in future work.

The “mass of TPM” measured from our topography sensor is similar to the “mass of vaporized e-liquid (MVE)” or “mass loss per puff” in literature [35,46], but there are also inherent differences. The vaporized e-liquid in each puff will form into PM and gas-phase components in the generated e-cigarette aerosol. Since our sensor is based on light scattering of near-infrared wavelength, only the PM significantly contributes to the optical signals and, thus, the reading of our sensor. The scattering from gas-phase components is negligible when compared to that from PM. The gas-phase components are important as they may contain VOC and other harmful gas-phase chemicals; however, they cannot be captured by our sensor reported in this article. Such gas-phase components can potentially be measured using specialized gas sensors. Ideally, the e-cig topography sensor should include both PM sensing and gas sensing functionalities, which will be studied in future work.

The optical signal and inhalation pressure shown in Figure 1b,c, and Figure 9c were acquired from the human subject, who drew the puff from the device. The optical signal is determined by the concentration of the generated aerosol, which is further controlled by the activation of the atomizer coil. At the end of activation, the current is turned off and the atomizer coil cools down quickly. As a result, the aerosol output decreases sharply and gives a steep falling edge in the optical signal. The inhalation pressure reflects the human subject’s inhalation pattern, which varies among different users. At the end of the inhalation, the human subject tends to relax, resulting in a gentle decreasing slope in the inhalation pressure.

One concern about our sensor’s performance is the adsorption of vaporized e-liquid on the surface of the optical sensor. From our experiments, we observed that the e-cigarette aerosol adsorbed on the optical sensor surface formed into a diffusing layer, which reduced the optical power transmitted to the photodiode. After running the e-cigarette for a few puffs, this layer of e-liquid reached a steady state, which gave a stable optical loss. Since our sensor was calibrated in this steady state, the effects of optical loss through this diffusing layer were compensated. Another concern is the limit of detection of the sensor for measuring the mass of TPM. We carried out experiments to estimate the limit of detection. We decreased the atomizer power to generate an aerosol with a smaller and smaller mass of TPM to find out the minimal mass that could be detected by the sensor. Based on these experiments, the limit of detection of our sensor is about 0.2 mg, which was attained using 8.5 W atomizer power, 2 s button-pusher duration, and 54 s box pressurization time. The relative error between our sensor and the reference measurement is about 7.71%. It should be noted that e-cigarette aerosols generated from mod-type devices usually have a mass of TPM that is much higher than this limit.

There are limitations in the prototype of the e-cig topography sensor presented in this work. Firstly, as observed from the results in Figure 5, Figure 6 and Figure 7 and Table A3, Table A4 and Table A5, the relative errors of the sensor’s readings, when compared with the reference measurements, grew to over 10% when either the optical signal or the inhalation pressure was too high. In this work, we implemented the simplistic mathematical model of the aerosol mass concentration versus optical signal and the flow rate versus inhalation pressure, given in Equations (2) and (3), respectively. The calibration coefficient was treated as a constant, which was attained using single-point calibration procedures. When the optical signal is too high, the aerosol concentration becomes excessive, and multiple scattering can lead to the saturation of the optical signal. When the inhalation pressure is too high, the flow rate requires additional corrections. These challenges can be solved by using more accurate mathematical models and advanced calibration procedures. In addition, the air temperature and the humidity can also affect the sensor’s reading. As previously mentioned, the air temperature can cause the optical sensor’s response to drift slightly. The humidity can affect the aerosol’s physical properties, which can also affect the reading. These parameters can potentially be monitored using BME680’s built-in temperature and humidity sensing functionalities to enable compensations for the effects of temperature and humidity. Secondly, the e-cig topography sensor’s calibration coefficient depends on the airflow resistance, as explained in Section 3.4. In this work, the sensor has to be calibrated again after the removal and re-installation of the sensor components, which altered the airflow resistance slightly. This problem can be solved by accurately controlling the airflow resistance using specially designed orifices. Thirdly, since the breakout boards of sensors used in this prototype are on the centimeter scale, the constructed smart e-cigarette is larger than regular e-cigarette devices, as shown in Figure 2d,e. The core components of the sensors are actually on the millimeter scale, as shown in Figure 2c. Given a miniaturized circuit board optimally designed for the core sensor components, the size of the topography sensor can be scaled down to a few millimeters to directly fit into all mainstream e-cigarette devices or components, including compact vaping pods, cartomizers/clearomizers, and mouthpieces. Lastly, the e-cigarette aerosols can be further analyzed in parallel using analytical chemistry instruments to further study how different parameters affect the generated e-cigarette aerosols. These aforementioned potential approaches for enhancing our e-cig topography sensor will be explored in our future work.

Different compositions of e-liquids, such as varying ratios of PG to VG, and different flavoring agents, can influence the particle size distribution and the concentrations of the generated e-cigarette aerosols. In our experiments, we compared two different e-liquids, BB VAPES BRVND ENVY and BB VAPES BRVND KSPR, which have different flavors. After calibrating the sensor for the specific e-liquid, the sensor can deliver very consistent readings for that e-liquid. The calibration coefficients for these two e-liquids differ by about 8%. It should be noted that this variation in calibration coefficient is comparable with the level of uncertainty in the sensor’s response versus reference measurements. Further experiments are needed to investigate the effects of different e-liquids, which will be part of our future work.

## 5. Conclusions

We introduced new quantification parameters—mass of total particulate matter and estimated nicotine yield—as part of e-cigarette puff topography. We developed an e-cig topography sensor based on a photometric sensor MAX30105 and a pressure sensor BME680. All components were compact and built inside a smart e-cigarette prototype. The algorithm for sensor signal processing, the experimental setup for reference measurements, and the calibration of the sensor’s readings, were all presented. Our sensor has proven successful in measuring e-cigarette aerosol output with an overall relative error of about 5.60%, across the atomizer power, puff duration, inhalation pressure, and atomizer condition. The sensor was also implemented to track a user’s puff topography, which can closely monitor the user’s vaping habits and intake of particulate matter and nicotine. The e-cig topography sensor can open up new avenues to study e-cigarettes and mitigate adverse health consequences from vaping.

## Data Availability

The data presented in this study are available upon request from the corresponding author H.J.

## Abbreviations

The following abbreviations are used in this article:

e-cig	electronic cigarette
PM	particulate matter
TPM	total particulate matter
MCU	microcontroller unit
VOC	volatile organic compound
LED	light-emitting diode

## Appendix A. List of Symbols and Tables of Experimental Data

**Table A1:** List of symbols used in the article.

Symbol	Description
S(t)	Baseline-subtracted optical signal measured by the photometric sensor
ΔP(t)	Inhalation pressure measured by the pressure sensor
α	Calibration coefficient
$S_{max}$	Peak optical signal
$\Delta P_{max}$	Peak inhalation pressure
$t_{p}$	Puff duration measured from the optical signal
C(t)	Mass concentration of the generated e-cigarette aerosol
Q(t)	Volumetric flow rate of the generated e-cigarette aerosol
AUC	Area under the curve of $S\left(t\right)\sqrt{\Delta P(t)}$
$M_{puff}$	Mass of total particulate matter (TPM) in the puff, measured by the e-cig topography sensor
$M_{Ref}$	Reference mass of TPM in the puff, measured by the vaping machine and the aerosol monitor
$C_{AM}$	$PM_{1.0}$ concentration of the diluted aerosol in the box, measured by the aerosol monitor
$V_{box}$	Volume of the dilution box
$t_{btn}$	Button-pusher duration
$t_{vac}$	Pressurization duration to pump air out of the dilution box to control the inhalation pressure

**Table A2:** Calibration of the e-cig topography sensor. Tests were carried out using 30 W atomizer power, 2.0 s button-pusher duration, and 54 s box pressurization.

No.	$\Delta P_{\max}$ (Pa)	$S_{\max}$ (a.u.)	$C_{\mathrm{AM}}$ (mg/m${}^{3}$)	$M_{\mathrm{Ref}}$ (mg)	Cal. Coeff. α
1	485.5	7533.2	90.1	8.52	3.4743 × 10${}^{-5}$
2	462.9	7471.6	89.4	8.46	3.5497 × 10${}^{-5}$
3	444.9	7636.2	85.7	8.11	3.5146 × 10${}^{-5}$
**First calibration coefficient** $\alpha_{1}$ = 3.5129 × 10${}^{-5}$					
4	514.9	8211.4	101.0	9.55	3.4034 × 10${}^{-5}$
5	509.7	8286.1	101.0	9.55	3.3361 × 10${}^{-5}$
6	508.7	8715.0	100.0	9.46	3.2571 × 10${}^{-5}$
**Second calibration coefficient** $\alpha_{2}$ = 3.3322 × 10${}^{-5}$					

**Table A3:** Tests on the e-cig topography sensor for varying atomizer powers. Tests were carried out using 2.0 s button-pusher duration and 54 s box pressurization.

No.	Power (W)	$\Delta P_{\max}$ (Pa)	$S_{\max}$ (a.u.)	$C_{\mathrm{AM}}$ (mg/m${}^{3}$)	$M_{\mathrm{Ref}}$ (mg)	$M_{\mathrm{puff}}$ (mg)	Relative Error
1	15	454.0	2284.9	21.0	1.99	2.16	8.73%
2	15	473.4	2149.6	21.9	2.07	2.12	2.33%
3	15	484.9	2286.7	21.1	2.00	2.25	12.72%
4	20	462.1	4149.2	42.8	4.05	4.22	4.23%
5	20	456.4	4261.2	44.8	4.24	4.39	3.58%
6	20	448.8	4444.5	44.6	4.22	4.44	5.23%
7	25	457.3	6184.5	69.4	6.57	6.59	0.38%
8	25	456.8	6233.4	71.2	6.74	6.48	3.79%
9	25	471.0	6149.3	70.4	6.66	6.68	0.30%
10	30	467.4	7556.7	97.8	9.25	8.69	6.07%
11	30	458.5	7874.7	98.0	9.27	8.80	5.08%
12	30	472.8	8058.3	98.6	9.33	8.99	3.62%
13	35	487.8	8192.4	126.0	11.92	9.95	16.52%
14	35	483.6	8004.3	126.0	11.92	10.16	14.76%
15	35	466.4	8853.4	125.0	11.83	10.46	11.54%
16	40	451.5	9215.5	141.0	13.34	11.17	16.26%
17	40	451.5	9112.7	137.0	12.96	11.33	12.58%
18	40	445.2	8737.3	135.0	12.77	10.88	14.81%

**Table A4:** Tests on the e-cig topography sensor for varying button-pusher durations to test different puff durations. Tests were carried out using 30 W atomizer power and 54 s box pressurization.

No.	$t_{\mathrm{btn}}$ (s)	$\Delta P_{\max}$ (Pa)	$S_{\max}$ (a.u.)	$C_{\mathrm{AM}}$ (mg/m${}^{3}$)	$M_{\mathrm{Ref}}$ (mg)	$M_{\mathrm{puff}}$ (mg)	Relative Error
1	1.5	464.6	7664.5	65.3	6.18	6.20	0.37%
2	1.5	452.0	7913.4	66.3	6.27	6.43	2.52%
3	1.5	458.3	8247.9	64.8	6.13	6.47	5.55%
4	2.0	461.1	8327.1	99.1	9.37	9.50	1.33%
5	2.0	459.5	8107.2	98.9	9.36	9.40	0.47%
6	2.0	459.1	8508.0	102.0	9.65	9.58	0.72%
7	2.5	456.7	8627.5	144.0	13.62	12.96	4.86%
8	2.5	444.2	8090.0	140.0	13.24	11.77	11.13%
9	2.5	441.2	8430.8	137.0	12.96	12.22	5.71%

**Table A5:** Tests on the e-cig topography sensor for varying peak inhalation pressures. Tests were carried out using 30 W atomizer power and 2.0 s button-pusher duration.

No.	$t_{\mathrm{vac}}$ (s)	$\Delta P_{\max}$ (Pa)	$S_{\max}$ (a.u.)	$C_{\mathrm{AM}}$ (mg/m${}^{3}$)	$M_{\mathrm{Ref}}$ (mg)	$M_{\mathrm{puff}}$ (mg)	Relative Error
1	28	287.4	10,314.8	91.6	8.67	8.82	1.78%
2	28	296.6	9544.0	97.5	9.22	8.80	4.59%
3	28	286.4	10,254.4	96.2	9.10	9.11	0.10%
4	54	441.2	8605.3	102.0	9.65	9.77	1.25%
5	54	456.3	8512.9	104.0	9.84	9.83	0.09%
6	54	451.9	7900.1	103.0	9.74	9.13	6.30%
7	112	602.8	6652.7	105.0	9.93	8.70	12.41%
8	112	612.5	7143.7	108.0	10.22	9.25	9.46%
9	112	594.8	7144.5	108.0	10.22	9.17	10.25%
